# Alloy Disordering Effects on the Thermal Conductivity and Energy Gap Temperature Dependence of Cd_1−x_Zn_x_Se Ternary Crystals Grown by the Bridgman Method

**DOI:** 10.3390/ma16113945

**Published:** 2023-05-25

**Authors:** Karol Strzałkowski, Ali Abouais, Amine Alaoui-Belghiti, Diksha Singh, Abdelowahed Hajjaji

**Affiliations:** 1 Institute of Physics, Faculty of Physics, Astronomy and Informatics, Nicolaus Copernicus University in Toruń, ul. Grudziądzka 5, 87-100 Toruń, Poland; 2 Engineering Science for Energy Lab, National School of Applied Sciences, Chouaib Doukkali University of El Jadida, El Jadida 24000, Morocco

**Keywords:** bulk crystal growth, II-VI alloys, lattice disorder, bowing parameter, thermal conductivity, CdZnSe crystals

## Abstract

Investigated in this work, Cd_1−x_Zn_x_Se-mixed ternary compounds were grown by the Bridgman method. Several compounds with zinc content varying in the range 0 < x < 1 were produced between two binary parents, CdSe and ZnSe crystals. Using the SEM/EDS technique, the accurate composition of formed crystals was determined along the growth axis. Thanks to that, the grown crystals’ axial and radial uniformity were determined. Characterization of the optical and thermal properties was undertaken. The energy gap was measured using photoluminescence spectroscopy for different compositions and temperatures. The bowing parameter describing the behavior of the fundamental gap with composition for this compound was found to be 0.416 ± 0.06. The thermal characteristics of grown Cd_1−x_Zn_x_Se alloys were systematically studied. The thermal diffusivity and effusivity of the crystals under investigation were experimentally determined, allowing the calculation of the thermal conductivity. We applied the semi-empirical model that Sadao Adachi developed to analyze the results. Thanks to that, it was possible to estimate the contribution arising from chemical disorder to the crystal’s total resistivity.

## 1. Introduction

Cd_1−x_Zn_x_Se n-type semiconductors received attention during the last few decades due to their wide applications in solar cells [1,2,3,4,5], photoelectrodes [6], photocatalysis [7], transistors [8], optical waveguides [9], sensors [10], light-emitting diodes (LED) [11], and lasers [12,13]. Their advantages are wide optical band gap, good stability, and full light spectrum coverage [14]. CdSe is an up-and-coming candidate for photoconductive and photoelectrochemical cells, whereas zinc selenide is an essential material for light-emitting devices. Nevertheless, photo corrosion was found when applying cadmium selenide in photoelectrochemical cells, whereas ZnSe was reported to be more stable though less photoactive due to its wide band gap [15,16,17]. Two binary CdSe and ZnSe crystals (parents) can be mixed to grow Cd_1−x_Zn_x_Se ternary alloys to overcome this shortcoming. This work evaluates the fundamental gap of the produced compounds with the composition.

Energy band gaps and lattice parameters in grown in this way alloys can be tuned according to the requirements, leading to new semiconductor compounds [18,19]. Such materials may be suitable for accomplishing the tasks of increased solar spectrum absorption and enhanced resistance toward photo corrosion. Investigating the thermal conductivity and diffusivity can help explain the thermal energy dissipation through these materials. This study aims to analyze the thermal characteristics (the thermal effusivity, diffusivity, and conductivity) of the mixed Cd_1−x_Zn_x_Se semiconductors with the composition from one binary compound to another (from CdSe to ZnSe). In order to carry this out, the photopyroelectric (PPE) technique in the front (FPPE) and back (BPPE) configurations were applied. Among other photothermal methods, such as photothermal radiometry [20,21], IR thermography or scanning thermal microscopy [22], and microphone detection, PPE is a contact technique. The thermal wave method can be used to estimate the thermal effusivity and diffusivity of studied samples by analyzing measured heat oscillations. Since thermal diffusivity, effusivity, and conductivity are related, it is possible to determine the thermal conductivity of the studied materials. The impact of composition on the thermal characteristics of particular crystals was presented and explored.

The critical property of materials used in various applications is their quality. Specifying the crystal’s lattice disorder is essential because the sensor’s efficiency and sensitivity depend on the material’s quality. It was shown how lattice disorder effects in the case of Cd_1−x_Zn_x_Se crystals affect the physical properties of mixed compounds. The results were analyzed using Sadao Adachi’s mixed crystals theoretical model [23]. Consequently, the lattice disorder’s contribution from mixing to the crystal lattice’s thermal resistivity was obtained.

## 2. Materials and Methods

### 2.1. Samples Preparation

Cd_1−x_Zn_x_Se-mixed alloys were grown by the high-pressure and high-temperature Bridgman–Stockbarger technique for different zinc concentrations (x = 0.05, 0.1, 0.5, 0.9, 0.95, and 1). An argon overpressure of about 150 bar was applied during the crystal growth. Stoichiometric mixtures of pure binary CdSe and ZnSe (6N, Koch-Light) powders were used as starting materials. Binary ZnSe and CdSe exhibited sphalerite (cubic, space group F-43m, a = b = c = 5.66 Å) and wurtzite (hexagonal, space group P6-3mc, a = b = 4.30 Å, c = 7.02 Å) crystal structure, respectively. A graphite crucible was filled with the powders, heated at about 1600 °C for a few hours, and then pulled down at a rate of 2.6 mm/h. More details on this procedure were published elsewhere [24].

**Figure 1:**
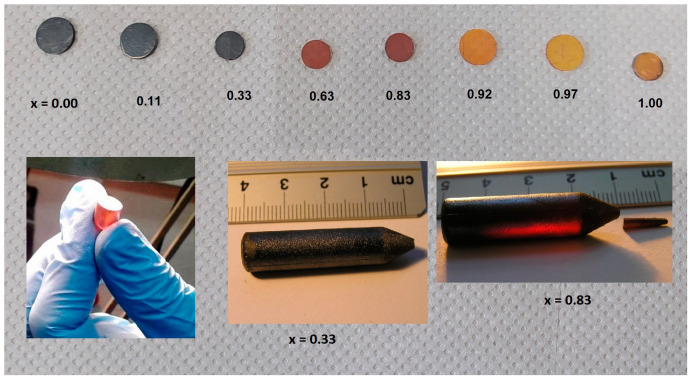
Examples of pictures of grown Cd_1−x_Zn_x_Se samples.

The obtained crystals were cylinders with raw dimensions of 8 to 10 mm in diameter and 40 to 50 mm in length. Grown in this way, ingots were cut perpendicular to the growth axis into about 1.2–1.5 mm plates. The samples were then mechanically ground (Al_2_O_3_ powder, 10 µm) and polished with fine powder (1 µm). In this work, the thermal properties of such prepared specimens (see Figure 1) were investigated. The samples for the photoluminescence measurements were additionally chemically etched in a solution of sulfuric acid (96%), potassium dichromate, and water.

**Figure 2:**
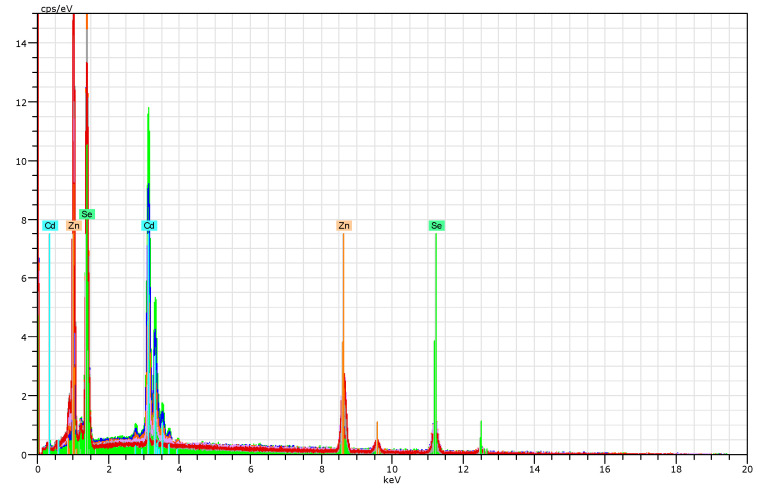
Selected spectra of characteristic radiation for Cd_1−x_Zn_x_Se samples under 20 keV excitation.

Using the SEM/EDS method, the accurate content of Cd, Zn, and Se elements was found. A Quantax 200 X-ray spectrometer and EDX XFlash 4010 detector with a 20 keV excitation energy were used to acquire the characteristic radiation. Examples of EDX spectra for 20 keV excitation for Cd_1−x_Zn_x_Se crystals are shown in Figure 2. Each sample was measured at three different points; this way, the radial uniformity of the plates was confirmed. The results shown in Table 1 were averaged over three points and represent atomic % of the zinc concentration in Cd_1−x_Zn_x_Se compounds. The atomic percentage of all elements was shown as well. The results presented in Table 1 show that measured composition generally followed a starting one. The most significant discrepancy for crystals with 63 and 83 percent of zinc content was observed. For other crystals, the difference was not substantial.

The thickness of each sample was measured at five different points using a micrometer with an accuracy of 10 µm. The results shown in Table 1 are averages, while standard deviation serves as the uncertainty. Such determined values were taken to calculate the thermal diffusivity of the investigated specimens.

### 2.2. Experimental Systems

**Figure 3:**
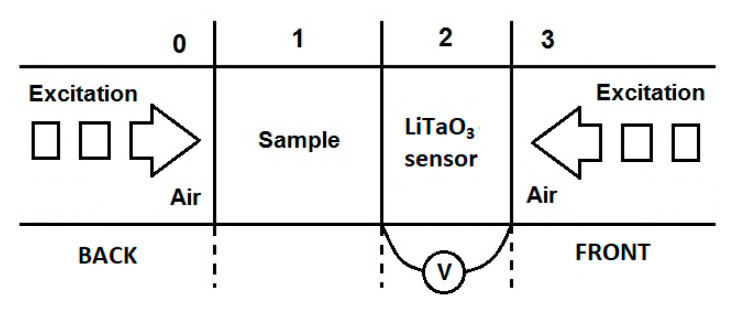
Back and front PPE detection configurations.

For thermal investigations, the photopyroelectric calorimetry method in different experimental configurations was used. An experimental setup consisted of a 0.5 mm pyroelectric detector LiTaO_3_ covered with thin layers of Cr and Au on both surfaces, an electronically modulated 300 mW power blue diode laser (Omicron, λ = 405 nm), and a two-phase lock-in amplifier SR850. The faces of the pyroelectric sensors were coated with opaque electrodes to absorb the incident light. The reference signal provided by the lock-in’s internal oscillator was used to modulate the incident radiation. An optically opaque sample was deposited on the sensor (see Figure 3). The sample was directly excited by the laser in the back detection configuration. The sensor detected the generated heat as it traveled through the plate. In this approach, the back configuration returned the thermal diffusivity of the specimen.

While in the front configuration, the sensor was directly exposed to laser radiation, and the specimen dissipated the heat. In this case, the measurement delivered the value of thermal effusivity. A small amount of ethylene glycol was a coupling liquid for excellent thermal contact between the pyro and the sample. The excitation light was modulation in the frequency range of 1 to 15 Hz and 5 to 45 Hz for the back and front modes, respectively. A normalizing procedure using an empty sensor was used for both BPPE and FPPE configurations [25]. The investigated specimens’ thermal characterization was computer-controlled and performed at ambient temperature. 

The typical photoluminescence setup was applied; it consisted of two lasers (He-Cd 325 nm, 30 mW, or 405 nm diode laser (Omicron) with output power up to 300 mW), a spectrometer (MicroHR Horiba Jobin Yvon), a helium cryostat (Advanced Research Systems), and a temperature controller (LakeShore 331). A filter wheel, thermoelectrically cooled CCD camera (Synapse Horiba Jobin Yvon, 1024 × 256 pixels), and diffraction gratings (1200 or 2400 lines/mm) were included in the spectrometer. All measurements were conducted at a temperature ranging from 9 K to room temperature. The resolution of spectra (single step) of the described setup was 0.14 nm.

### 2.3. Basic Theory of PPE

The photothermal techniques were based on generating a temperature field in the specimen due to the absorption of electromagnetic radiation; this process depended on the specimen’s optical and thermal properties. The photopyroelectric method can determine the thermal diffusivity using the phase-lag technique [25,26]. The following expression describes the normalized phase *Θ_n_* as a function of modulation frequency *f* under the assumption of one-dimensional heat transport through the sample and ideal thermal contact between the specimen and the pyro:

(1) $$\Theta_{n}=\Theta_{0}-L_{s}{\left(\frac{\pi f}{\alpha_{s}}\right)}^{1/2}$$

where *α_s_* and *L_s_* are the thermal diffusivity and thickness of the sample, respectively. The thermal diffusivity of the specimen can be extracted from the slope *a* of the phase-frequency graph according to the formula [25,26]:

(2) $$\alpha_{s}=\frac{L_{s}^{2}\pi }{a^{2}}$$

The thermal effusivity of the specimen can be measured in the FPPE configuration. In this case, the definition of the expression for the normalized phase is [25,26]:

(3) $$\Theta_{n}=arctan\frac{\left(1+R_{sp})e^{-a_{p}L_{p}}sin(a_{p}L_{p}\right)}{1-(1+R_{sp})e^{-a_{p}L_{p}}cos(a_{p}L_{p})}$$

where *e* is the thermal effusivity and *b_sp_* = *e_s_*/*e_p_*, *R_sp_* = (*b_sp_* − 1)/(*b_sp_* + 1) is the thermal wave reflection coefficient at the sample/pyro contact, *L_p_* is the thickness of the sensor, *a_p_* is the reciprocal of the thermal diffusion length *µ_p_* where *a_p_* = 1/*µ_p_*, *µ_p_* = (2*α_p_*/ω)^1/2^, and *ω* is the angular modulation frequency.

All thermal parameters were interrelated. The following formula can be taken to determine the thermal conductivity *k* of the specimen [25]:

(4) $$k=e\alpha^{1/2}$$

## 3. Experimental Results and Discussion

### 3.1. Photoluminescence Characterization

**Figure 4:**
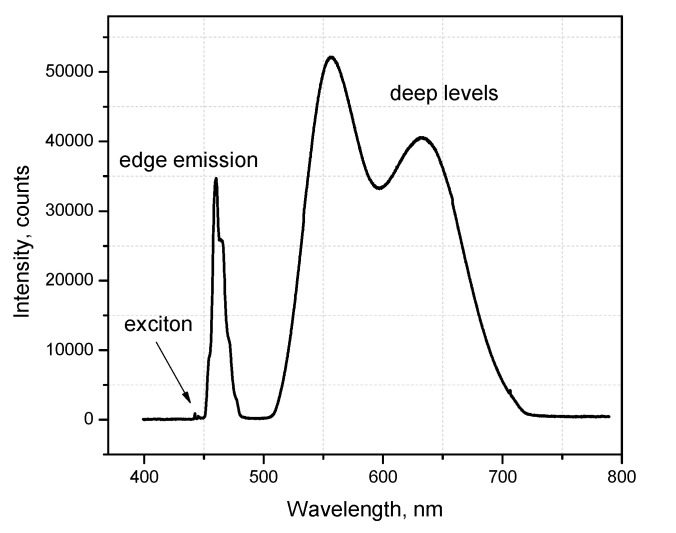
The emission spectrum of the ZnSe crystal at 10 K.

Figure 4 presents the photoluminescence spectrum of the ZnSe binary crystal at low temperatures. Typically, it consisted of a few emissions: high energy edge luminescence with visible phonon replicas, broad bands connected with deep levels, and violet-blue excitonic emission.

For high crystalline quality, one can investigate the temperature evolution of the excitonic emission, particularly once the bound energy of the electron-hole pair was high enough (more than 10 meV). Both parents ZnSe and CdSe fulfilled this condition, and we can determine the excitonic energy gap at ambient temperature. It should be considered that such a determined band gap value is slightly smaller than the real one due to the bound energy of the excitons [27]. In the case of mixed Cd_1−x_Zn_x_Se compounds, this value was not higher than 20 meV (binding energy of the excitons in binary ZnSe crystal).

**Figure 5:**
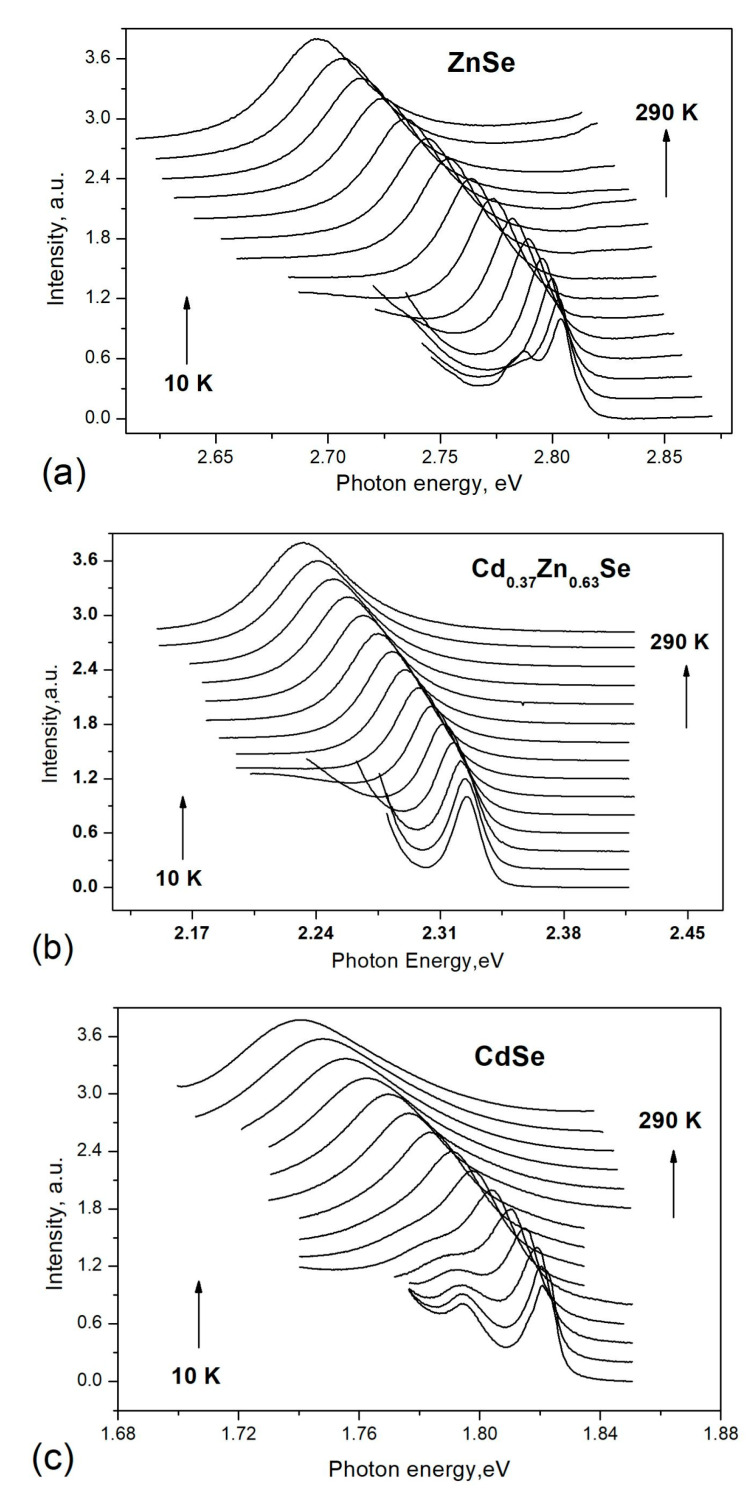
The temperature evolution of the excitonic emission of ZnSe (**a**), Cd_0.37_Zn_0.63_Se (**b**), and CdSe (**c**) crystals.

Figure 5 presents the temperature evolution of the excitonic emission for selected crystals: ZnSe (a), Cd_0.37_Zn_0.63_Se (b), and CdSe (c). Two maxima at low temperatures were observed in the spectrum for both binary semiconductors. A temperature broadening of the first maximum was seen simultaneously with a monotonic shift to lower energies. This was typical behavior for the recombination of free excitons. The second peak was thermally quenched around 70 and 90 K, probably related to a bound exciton. Recombination of only free excitons was observed for all grown mixed ternary Cd_1−x_Zn_x_Se crystals.

The excitonic emission was recordable for all crystals up to room temperature. Therefore, one can conclude the crystals exhibited good quality. Thanks to that, the Varshni formula was used to interpret the temperature evolution *f* the excitonic emission [28]:

(5) $$E_{g}(T)=E_{g}(0)-\frac{\gamma \cdot T^{2}}{\beta +T}$$

where *Eg*(0) is the band gap value at 0 K, *γ* and *β* constants are, respectively connected with the electron (exciton)—phonon interaction and Debye temperature.

**Figure 6:**
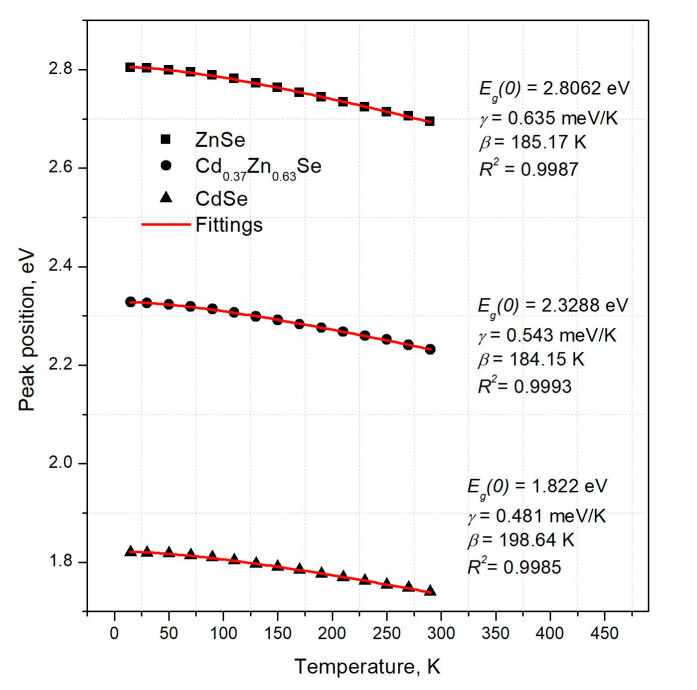
Temperature evolution of the excitonic emission with theoretical fitting (red line) performed with Equation (5) for ZnSe (squares), Cd_0.37_Zn_0.63_Se (circles), and CdSe (triangles) crystals.

Figure 6 illustrates the maximum excitonic emission’s temperature dependency with the fitting of Equation (5) for different crystals (R^2^ better than 0.998). In the case of pure ZnSe and CdSe crystals, the parameters *γ* and *β*, obtained by the fitting agreed with the literature data [29]. All obtained parameters are presented in Table 2. 

The spectra of the examined crystals were obtained for a monochromator slit of 0.1 mm or less and with a spectral resolution of 0.15 nm. The precision of determining the position of excitonic emission at RT was around 2–3 meV. Therefore, such a value served as the uncertainty of the excitonic energy gap. The behavior of the energy gap as a function of the composition for Cd_1−x_Zn_x_Se ternary alloys can be described by:

(6) $$E_{Cd{\;}_{1-x}Zn_{x}Se}(x)=xE_{ZnSe}+\left(1-x\right)E_{CdSe}+x\left(x-1\right)b$$

where *E_ZnSe_* and *E_CdSe_* are fundamental band gap values for binary *ZnSe* and *CdSe* semiconductors, respectively, and *b* is the parameter describing the bowing.

**Figure 7:**
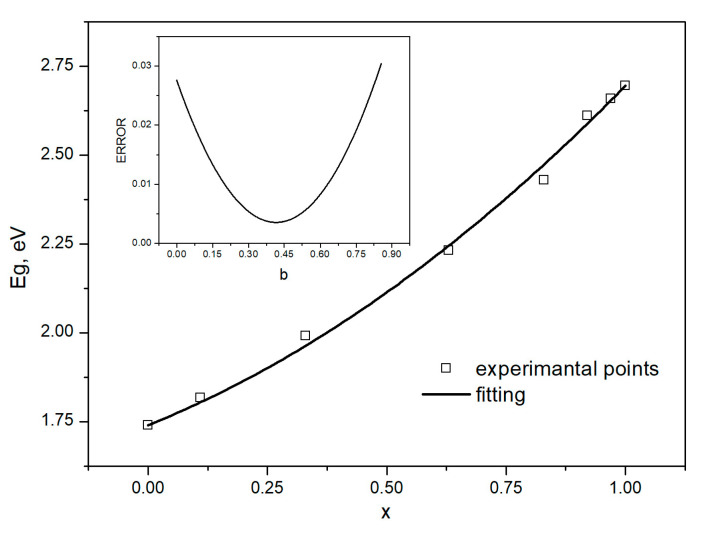
The behavior of the energy gap as a function of zinc content *x* for Cd_1−x_Zn_x_Se crystals, squares are experimental points, the line is fitting of Equation (6), and the error resulting from the fitting is indicated in the box, where *b* is the bowing parameter.

Figure 7 presents the measured fundamental gap values of the Cd_1−x_Zn_x_Se alloys versus zinc content *x*. The squares correspond to the determined points, and the line represents the best fitting of Equation (6) with a determination coefficient of *R^2^* = 0.9965. The bowing parameter value *b*, determined to be 0.416 ± 0.06, corresponded precisely to the minimal error (also shown in Figure 6). This value was very similar to the findings and results of other researchers. Mourad et al. [30] obtained an equal value (0.41 ± 0.09) of the bowing parameter for epitaxial layers of Cd_1−x_Zn_x_Se alloys. Venugopal et al. determined the *b* parameter of Cd_1−x_Zn_x_Se bulk-like nanowires at room temperature as 0.45 ± 0.02 [31]. 

### 3.2. Thermal Results

**Figure 8:**
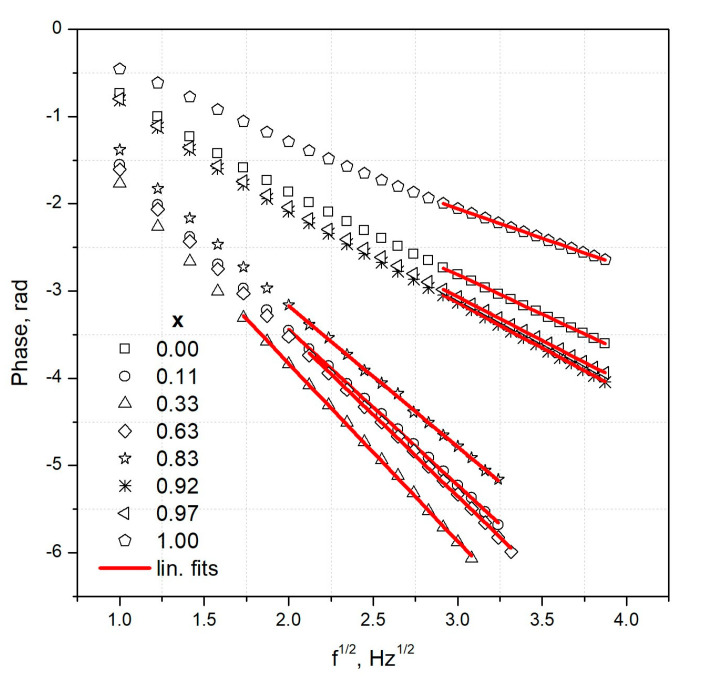
Phase characteristics measured in the back configuration of Cd_1−x_Zn_x_Se crystals for all zinc concentrations versus square root of the frequency, points represent the experiment, and lines are fittings obtained with the least square method.

The phase dependences of the BPPE signal as a function of the square root of the frequency for all Cd_1−x_Zn_x_Se crystals are presented in Figure 8. For the low frequencies, the sample and the sensor were thermally thin, and so, the nonlinear behavior of the phase was seen. Consequently, linear fits using the least square method were performed from 3–8 Hz, where the sensor and the specimen were thermally thick. Different frequency fitting range depended on the thermal diffusivity of the sample. The ZnSe crystal exhibited the highest thermal diffusivity, whereas the Cd_0.37_Zn_0.63_Se sample had the lowest value. Consequently, the first specimen started to be thermally thick from 8 Hz and the latter from 3 Hz. The fitting quality was better than 0.9999 in every case, according to the statistical determination coefficient *R^2^*. We calculated the samples’ thermal diffusivity according to Equation (2).

**Figure 9:**
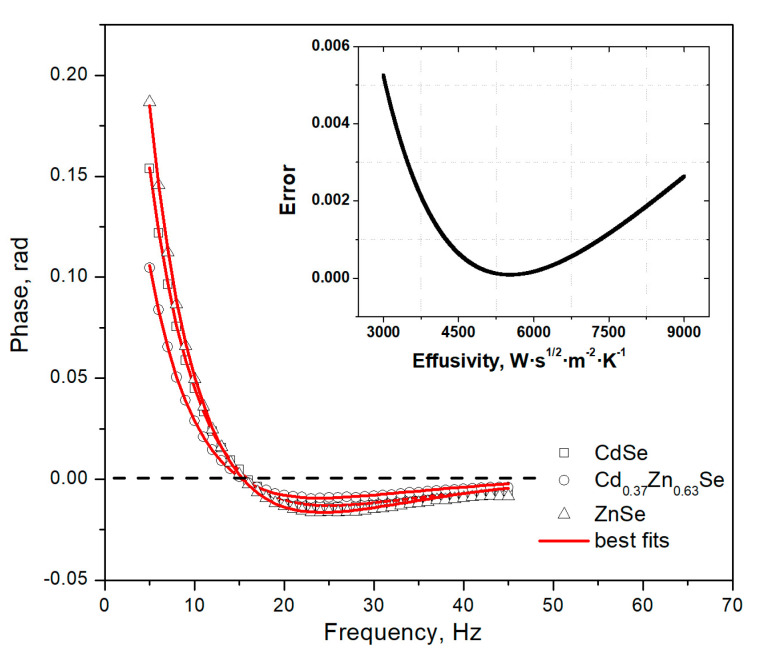
PPE phase of selected ZnSe (triangles), CdSe (squares), and Cd_0.37_Zn_0.63_Se (circles) crystals versus frequency points refer to the measurement. Lines are the best fittings obtained with Equation (3). The fitting error for the ZnSe crystal is shown in the inset.

The front measurement configuration (FPPE technique) was used to measure the thermal effusivity of the specimens. The modulation frequency was changed in the 5–45 Hz range, resulting in the sensor and sample satisfying both thermal thickness regimes. In Figure 8, the phase characteristics of Cd_1−x_Zn_x_Se samples versus frequency are presented; the lines are the best fits obtained using Equation (3) and the least squares method, while the points represent the measured values. The results are given for three selected samples. The determination coefficient was around 0.999, a bit lower than in the BPPE. The error of the fitting procedure for the ZnSe crystal is shown in the inset of Figure 9. The thermal effusivity value of the investigated specimen can be derived from the minimum observed in the error graph. The curves cross zero phase points for a similar frequency. According to the PPE theory, this proves that measurement and normalization procedures were carried out correctly. For the calculations of thermal diffusivity in the front configuration, one should use the sensor’s thermal parameters (*𝛼_𝑝_* =1.36 × 10^−6^ m^2^·s^−1^ and *𝑒_𝑝_* = 3660 W m^−2^∙s^1/2^∙L^−1^ [25,26]). 

### 3.3. Lattice Thermal Resistivity

Using Equation (4), one may determine the thermal conductivity of specimens after determining their thermal diffusivity and effusivity. Table 3 summarizes all thermal parameters of the Cd_1−x_Zn_x_Se crystals for different zinc (x) content. We averaged three measurements to obtain the thermal diffusivity and effusivity values, and the standard deviation served as an uncertainty. At the same time, the total differential approach was used to estimate the thermal conductivity error. Table 3 shows high experimental repeatability by proving that the uncertainties were 1% or less than the measured value. The first source of the uncertainty was the accuracy of the micrometer (10 µm) used to determine the thickness. The coupling fluid between the sample and the sensor was the most significant error source. However, previous studies found that the influence of the fluid can be minimized and was in the range of 2–3% [32]. All sources together resulted in the final uncertainty of 4–5%.

The thermal parameters presented in Table 3 behaved similarly in the composition’s function. CdSe and ZnSe crystals exhibited better thermal conductivity when compared with Cd_0.08_Zn_0.92_Se or Cd_0.03_Zn_0.97_Se mixed crystals. However, in the case of the latter one, the difference with CdSe was relatively small. 

When the lattice played a significant role in heat transport, this was the typical and expected behavior of the crystals. All thermal parameters of the specimens decreased when zinc and cadmium were mixed. This effect was caused mainly by the components’ different atomic radii, introducing the disorder into the crystal. Previous studies on Cd_1−x_Zn_x_Te [33], Zn_1−x_Be_x_Se, and Zn_1−x_Mg_x_Se [25] mixed crystals showed similar behavior.

As deducted, the main contribution to the lattice’s thermal conductivity is related to a phonon mechanism in our case [34]. This was expected since we were working with II–VI wide-band semiconducting crystals. In the case of semiconductor alloys, lattice thermal conductivity required considering a contribution resulting from a random distribution of constituent atoms in the crystal host. A model describing the behavior of the lattice thermal conductivity for mixed crystals was first proposed by Abeles [35]. Later, Adachi [23] described the thermal resistivity 𝑊(𝑥) of the ternary A_1−x_B_𝑥_C system by a simple formula:

(7) $$W(x)=xW_{AC}+(1-x)W_{BC}+x(1-x)C_{A-B}$$

where *W_AC_* and *W_BC_* are binary thermal resistivities, and *C_A−B_* is a contribution from the lattice disorder. One can quickly transfer Equation (7) into lattice thermal conductivity *K*(*x*):

(8) $$K(x)=\frac{1}{W(x)}=\frac{1}{xW_{AC}+(1-x)W_{BC}+x(1-x)C_{A-B}}$$

**Figure 10:**
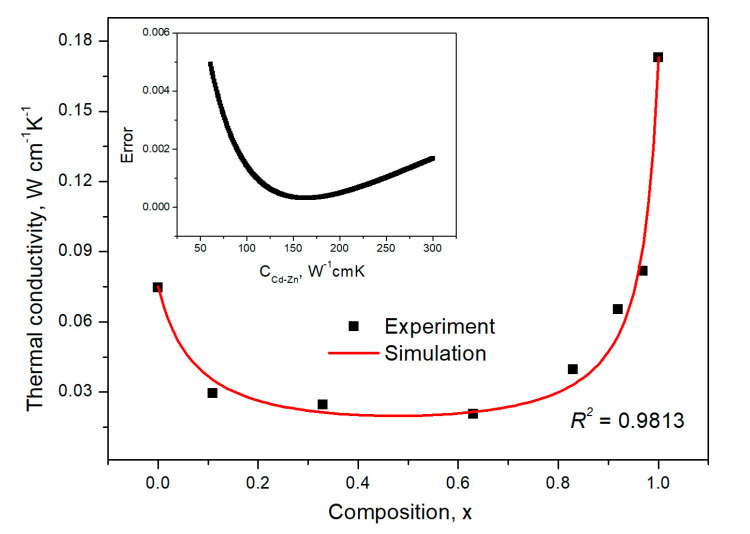
The thermal conductivity of the Cd_1−x_Zn_x_Se-mixed crystals versus Zinc content *x*. The points are measured data, and the solid red line is best fitting obtained by applying Equation (8). The fitting error is shown in the inset.

Figure 10 presents the thermal conductivity for Cd_1−x_Zn_x_Se alloys versus Zinc content x. The experimental points represent values from Table 3, and the red line is the least squares fitting to the theoretical values of Equation (8). It was found that the statistical determination coefficient was 0.9813. Figure 9 shows that the dependence of the thermal conductivity versus composition had a minimum in the middle (at x = 0.5). The most significant changes occurred at the curve’s edges, and a plateau was in the middle. From the fitting, it was determined that the chemical disorder contributed 162.8 ± 16.5 W^−1^·cm·K to the total thermal resistivity of the Cd_1−x_Zn_x_Se alloy. This value was the highest compared to the results obtained for other mixed II-VI ternary compounds. In the case of Zn_1−x_Mg_x_Se, Zn_1−x_Be_x_Se, and Cd_1−x_Zn_x_Te systems, the contribution to the thermal resistivity was determined between 116 W^−1^·cm·K and 139 W^−1^·cm·K [25,35]. On the other hand, compared to typical III-V crystals, the results obtained were a few times larger [23]. However, for both classes of semiconductors, the character of the thermal conductivity behavior was similar.

## 4. Conclusions

The Bridgman technique was used to grow binary parent crystals of CdSe and ZnSe and mixed ones. These alloys’ thermal and optical properties were investigated. The composition and uniformity were determined by applying the SEM/EDS method in the first stage. The results confirmed that the measured element content generally followed the starting one. The excitonic emission was present and recordable up to ambient temperature for all crystals. The energy gap of the investigated Cd_1−x_Zn_x_Se crystals was accurately obtained from photoluminescence spectroscopy. The quadratic equation described how the band gap energy changed with the composition. Thanks to that, the bowing parameter *b* value was found (0.416 ± 0.06). In comparison with the literature, the obtained value was in good agreement. The energy gap and the lattice constant of the binary semiconductors were fixed. Mixing of the CdSe and ZnSe binary parent crystals allowed tuning of the lattice constant and energy gap in the range from 1.74 to 2.7 eV. Thanks to that, both parameters can be adjusted according to the application requirements. Using the PPE method in the front and back configurations, a complete thermal characterization of the Cd_1−x_Zn_x_Se crystals was conducted. The experiment’s thermal diffusivity and effusivity values were used to calculate the thermal conductivity of the studied semiconductors. It turned out that mixing CdSe and ZnSe leads to decreasing all thermal parameters of the specimens. The thermal results were verified using the Adachi formula given for ternary materials. Thanks to that, the contribution from the lattice disorder to the total resistivity of the crystal was determined as *C_Cd_*_−*Zn*_ = 162.8 ± 16.5 W^−1^·cm·K.

## Figures and Tables

**Table 1:** The composition and the thickness of grown Cd_1−x_Zn_x_Se samples.

Starting x, %	Measured x, %	Cd Atomic%	Zn Atomic%	Se Atomic%	Thickness mm
0	-	-	-	-	1.15 ± 0.01
10	11 ± 0.5	42.37 ± 0.19	5.49 ± 0.27	52.14 ± 0.36	1.38 ± 0.09
30	33 ± 0.2	32.24 ± 0.72	16.35 ± 0.52	51.41 ± 1.26	1.33 ± 0.02
50	63 ± 0.5	17.72 ± 0.41	31.01 ± 0.95	52.27 ± 1.21	1.13 ± 0.03
70	83 ± 0.4	8.24 ± 0.28	41.32 ± 0.88	51.02 ± 1.01	1.35 ± 0.02
90	92 ± 0.4	4.38 ± 0.18	45.89 ± 1.05	50.54 ± 2.29	1.12 ± 0.02
95	97 ± 0.3	1.74 ± 0.22	48.64 ± 0.88	51.24 ± 1.09	1.24 ± 0.08
100	-				1.09 ± 0.01

**Table 2:** Excitonic energy gap at RT of Cd_1−x_Zn_x_Se crystals *γ* and *β* parameters.

*x*	*E_g_* at RT eV	*γ* meV·K^−1^	*γ* K	*R^2^* -
0	1.740 ± 0.003	0.504 ± 0.025	220.13 ± 25.32	0.99850
11	1.817 ± 0.003	0.360 ± 0.003	60.49 ± 3.42	0.99988
33	1.991 ± 0.003	0.375 ± 0.013	75.03 ± 8.71	0.99941
63	2.232 ± 0.003	0.551 ± 0.013	190.23 ± 11.78	0.99930
83	2.430 ± 0.003	0.549 ± 0.001	95.34 ± 13.31	0.99975
92	2.611 ± 0.003	0.525 ± 0.018	160.17 ± 24.46	0.99844
97	2.658 ± 0.003	0.583 ± 0.021	199.69 ± 17.41	0.99956
100	2.695 ± 0.003	0.635 ± 0.037	185.17 ± 27.71	0.99870

**Table 3:** Thermal diffusivity, effusivity, and conductivity of the Cd_1−x_Zn_x_Se alloys.

x	Thermal Diffusivity (m^2^·s^−1^) × 10^−6^	Thermal Effusivity (W·s^1/2^·m^−2^·K^−1^)	Thermal Conductivity (W·cm^−1^·K^−1^) × 10^2^
0	4.981 ± 0.098	3359.7 ± 15.9	7.498 ± 0.109
0.11	1.847 ± 0.050	2163.3 ± 2.0	2.939 ± 0.043
0.33	1.420 ± 0.035	2055.0 ± 13.2	2.449 ± 0.046
0.63	1.148 ± 0.031	1923.3 ± 83.3	2.060 ± 0.117
0.83	2.237 ± 0.045	2638.3 ± 28.4	3.946 ± 0.082
0.92	3.623 ± 0.045	3423.3 ± 23.1	6.516 ± 0.085
0.97	4.835 ± 0.007	3714.0 ± 68.5	8.067 ± 0.157
1	9.853 ± 0.084	5515.0 ± 136.1	17.312 ± 0.501

## Data Availability

All data are fully available without restriction. The datasets used and/or analyzed during the current study are available from the corresponding author upon reasonable request.
